# Vancomycin-conjugated polydopamine-coated magnetic nanoparticles for molecular diagnostics of Gram-positive bacteria in whole blood

**DOI:** 10.1186/s12951-022-01606-3

**Published:** 2022-09-05

**Authors:** Abdurhaman Teyib Abafogi, Tepeng Wu, Daekyu Lee, Jinyeop Lee, Gyoujin Cho, Luke P. Lee, Sungsu Park

**Affiliations:** 1 School of Mechanical Engineering, Sungkyunkwan University (SKKU), Seobu-ro 2066, Jangan-gu, Suwon, 16419 Korea; 2 KingoBio Inc., 31 Digital-ro 30-gil, Guro-gu, Seoul, 08390 Korea; 3 Department of Biophysics, Institute of Quantum Biophysics, Sungkyunkwan University, Suwon, 16419 Korea; 4 Department of Medicine, Harvard Medical School, Brigham Women’s Hospital, Boston, MA 02115 USA; 5 Department of Bioengineering, Department of Electrical Engineering and Computer Science, University of California at Berkeley, Berkeley, CA 94720 USA

**Keywords:** Sepsis, Immunomagnetic separation, Preconcentration, Polydopamine, Molecular diagnostics, Aggregation

## Abstract

**Background:**

Sepsis is caused mainly by infection in the blood with a broad range of bacterial species. It can be diagnosed by molecular diagnostics once compounds in the blood that interfere with molecular diagnostics are removed. However, this removal relies on ultracentrifugation. Immunomagnetic separation (IMS), which typically uses antibody-conjugated silica-coated magnetic nanoparticles (Ab-SiO_2_-MNPs), has been widely applied to isolate specific pathogens in various types of samples, such as food and environmental samples. However, its direct use in blood samples containing bacteria is limited due to the aggregation of SiO_2_-MNPs in the blood and inability to isolate multiple species of bacteria causing sepsis.

**Results:**

In this study, we report the synthesis of vancomycin-conjugated polydopamine-coated (van-PDA-MNPs) enabling preconcentration of multiple bacterial species from blood without aggregation. The presence of PDA and van on MNPs was verified using transmission electron microscopy, X-ray photoelectron spectroscopy, and energy disruptive spectroscopy. Unlike van-SiO_2_-MNPs, van-PDA-MNPs did not aggregate in the blood. Van-PDA-MNPs were able to preconcentrate several species of Gram-positive bacteria in the blood, lowering the limit of detection (LOD) to 10 colony forming units/mL by polymerase chain reaction (PCR) and quantitative PCR (qPCR). This is 10 times more sensitive than the LOD obtained by PCR and qPCR using van-SiO_2_-MNPs.

**Conclusion:**

These results suggest that PDA-MNPs can avoid aggregation in blood and be conjugated with receptors, thereby improving the sensitivity of molecular diagnostics of bacteria in blood samples.

**Supplementary Information:**

The online version contains supplementary material available at 10.1186/s12951-022-01606-3.

## Background

Sepsis is a life-threatening immune response to bloodstream bacterial infections [1, 2]. In 2017, about 48.9 million people were diagnosed with sepsis, while about 11 million worldwide died due to sepsis [2]. Unless it is detected early and timely interventions are applied, sepsis mortality increases up to 10% per hour [3]. Since the standard diagnostic method of a blood culture takes 24–48 h for detection [4], broad-spectrum antibiotics must be used to treat patients without identification of the pathogens [3]. This reduces the effectiveness of treatment and increases the likelihood of antibiotic resistance [3]. Molecular diagnostics such as polymerase chain reaction (PCR) reduce detection times to 2 h [5]. However, endogenous inhibitory compounds such as heme and leukocyte DNA and anticoagulants such as EDTA and heparin [6] negatively affect PCR sensitivity [7]. Therefore, these inhibitory compounds should be removed from the sample prior to PCR [7].

Various techniques have been developed to isolate pathogens from samples, including filtration [8], centrifugation and sedimentation [9], inertial separation [10], and immunomagnetic separation (IMS) [11]. Among them, IMS is the most sensitive because it uses a target-specific antibody [12] conjugated to superparamagnetic silica-coated magnetic nanoparticles (SiO_2_-MNPs) to capture a target pathogen through antigen–antibody interaction. However, when the silica surface is exposed to air or water, an oxide layer is formed on the surface of the silica tetrahedral crystals (silanol groups) [13]. Then, reactive oxygen species and reactive moieties of the silanol group interact with cell membranes [14], causing non-specific adsorption of blood cells and platelets. This adsorption causes aggregation of SiO_2_-MNPs and promotes the transfer of residual inhibitory compounds to the DNA extraction step. These compounds then inhibit PCR amplification [7, 15]. Therefore, to improve the sensitivity of PCR for the detection of sepsis, effort should be focused on the development of a surface coating method of MNPs that can reduce non-specific adsorption of blood cells and platelets.

Polydopamine (PDA) can prevent the non-specific binding of blood cells and platelets to micro/nanoparticles due to their strong hydrophilicity [16, 17]. It is a highly adaptable polymer that can be used to coat a variety of materials in a single step through the oxidative self-polymerization of dopamine [18, 19]. Because of its reactivity with amine and thiol functional groups, it can also be used to immobilize biomolecules on surfaces [18, 20, 21]. For example, a PDA coating has been used to immobilize antimicrobial imidazolium-based ionic liquid to MNPs to remove bacteria from the blood [17]. Similarly, PDA has been used to coat MNPs to functionalize probe oligonucleotides [22]. However, PDA-MNPs have not been used to enrich bacteria in blood to improve the sensitivity of molecular diagnostics.

To isolate a broad range of bacterial species, antibiotics can be conjugated to MNPs instead of species-specific antibodies [23, 24]. Among antibiotics, vancomycin (van) has been conjugated to MNPs to isolate Gram-positive bacteria [24]. Van is a bacteriostatic antibiotic that inhibits bacterial cell wall synthesis by binding to the d-alanyl-d-alanyl terminus of the peptidoglycan layer of Gram-positive bacteria through a hydrogen bonding [25, 26]. Therefore, it has great potential for use in the separation of Gram-positive bacteria [23, 24]. For example, van-MNPs were used to separate Gram-positive bacteria such as *Staphylococcus aureus* from whole blood and improved the sensitivity of quantitative PCR (qPCR) to about 5 colony forming units (CFU) per 1 mL of blood. However, the method still required the removal of blood cells using centrifugation due to their fouling with blood cells [24].

**Fig. 1:**
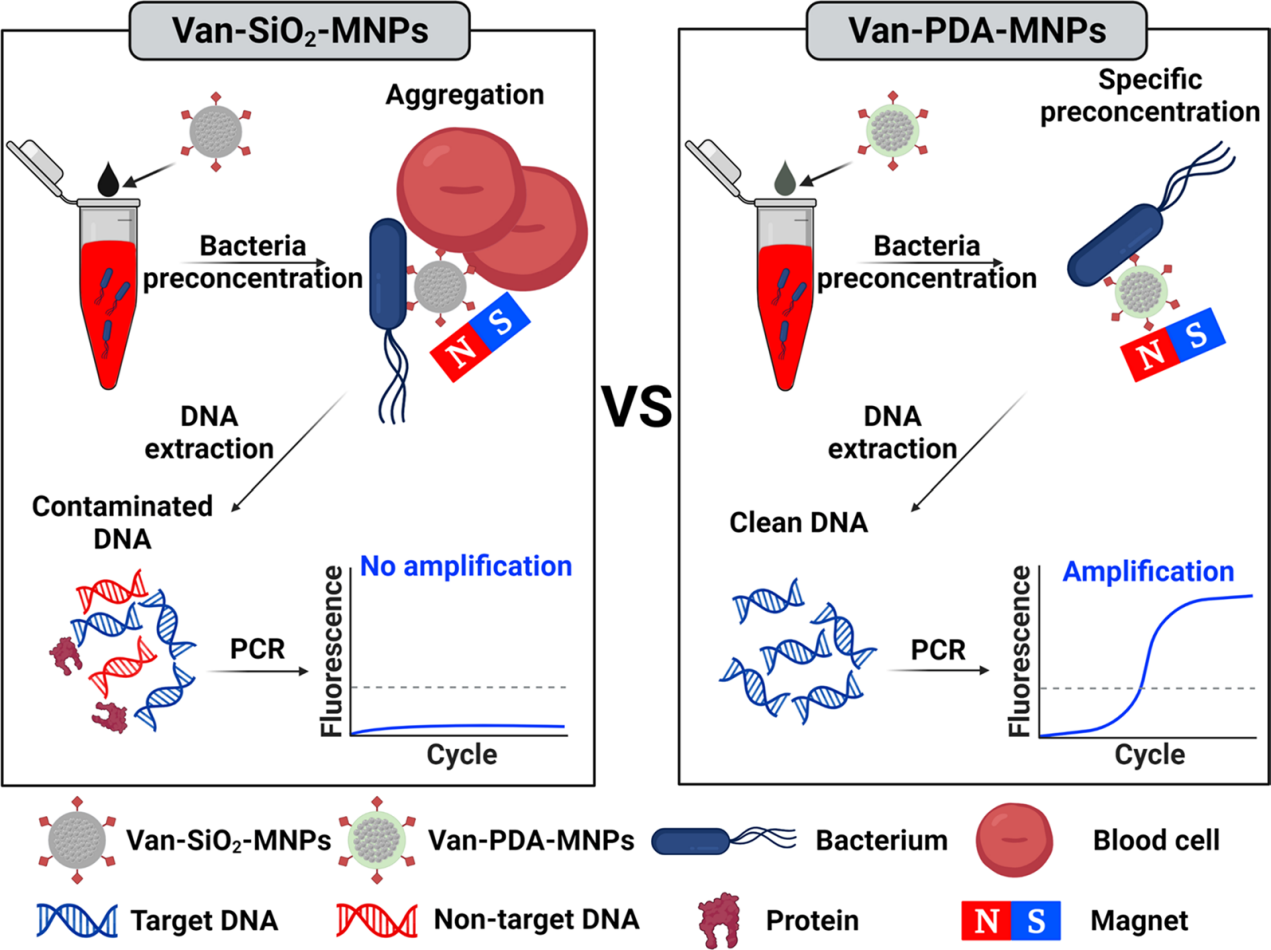
Schematics illustrating bacterial preconcentration without non-specific aggregation in the blood using vancomycin-conjugated polydopamine-coated magnetic nanoparticles (van-PDA-MNPs) instead of vancomycin-conjugated silica-coated magnetic nanoparticles (van-SiO_2_-MNPs) for sepsis molecular diagnostics. www.biorender.com was used to make the schematics

In this study, we report the synthesis of PDA-MNPs conjugated with van (van-PDA-MNPs), enabling the preconcentration of multiple bacterial species from blood without aggregation. The presence of PDA and van in the MNPs was verified using transmission electron microscopy (TEM), energy dispersive spectroscopy (EDS) mapping, zeta-potential measurement, and X-ray photoelectron spectroscopy (XPS) analysis. The advantages of van-PDA-MNPs were demonstrated by comparing the aggregation and bacterial preconcentration of van-PDA-MNPs and van-SiO_2_-MNPs in whole blood samples (Fig. 1). The feasibility of van-PDA-MNPs for molecular detection of Gram-positive bacteria in blood was tested with whole blood samples spiked with multiple species of Gram-positive bacteria and methicillin-resistant *S. aureus* (MRSA). Van-PDA-MNPs can preconcentrate these microorganisms up to 100-fold in the blood within 30 min, lowering the limit of detection (LOD) to 10 CFU/mL by PCR and qPCR.

## Results and discussion

### Characterization of van-PDA-MNPs

**Fig. 2:**
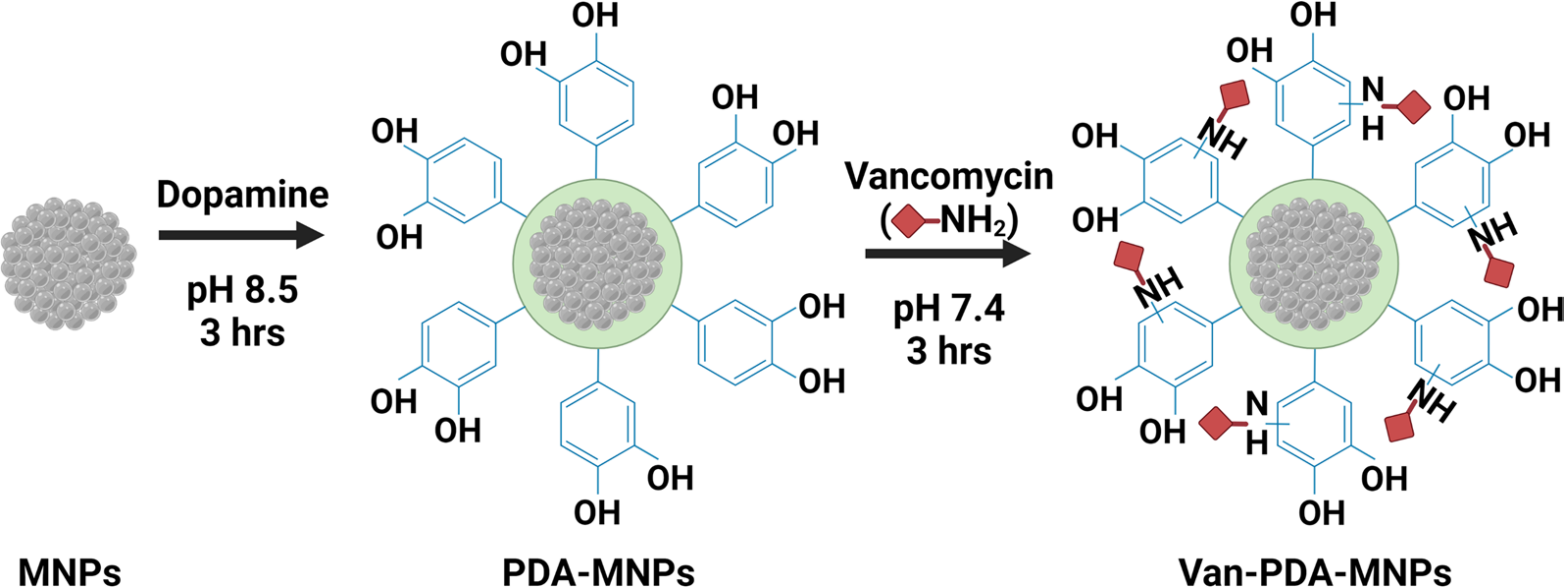
The schematic represents the synthesis of PDA-MNPs and van-PDA-MNPs. Freshly prepared MNPs form larger MNPs through dopamine coating. Van was grafted to PDA-MNPs through the reaction of primary amino groups of van to phenyl rings of PDA. www.biorender.com was used to make the schematic

**Fig. 3:**
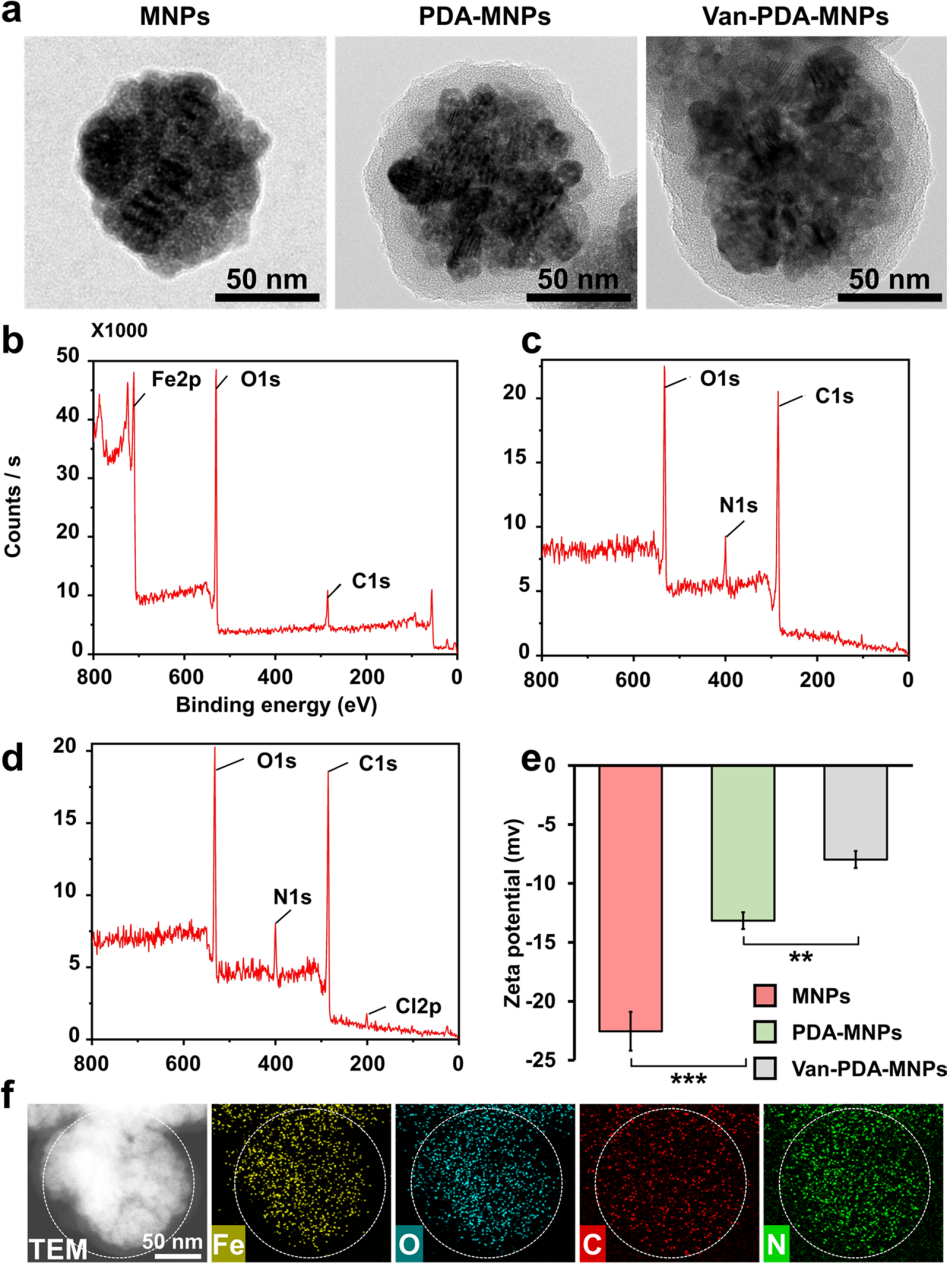
Characterization of iron oxide MNPs_,_ PDA-MNPs, and van-PDA-MNPs. **a** Images of each type of MNP on a 300-mesh copper grid were obtained by TEM (JEM-2100F) (JEOL Ltd.). Element composition in **b** MNPs, **c** PDA-MNPs and **d** van-PDA-MNPs using XPS. Each type of MNP was placed on a glass slide, and their elemental composition was studied by exciting the MNPs to mono-energetic Al kα x-rays and measuring the energy of photoelectrons emitted by electron energy analyser (ESCALAB250 XPS analyser). **e** The zeta potential of each type of MNP was measured using a Zetasizer Nano ZS (Malvern Instruments). **f** Elemental mapping of van-PDA-MNPs obtained by TEM with EDS. Student’s *t*-test, ***: *P* < 0.001. **: *P* < 0.01.*: *P* < 0.05. n = 3

For the synthesis of van-PDA-MNPs, freshly prepared MNPs were first coated with PDA, and van was then grafted onto PDA-MNPs through the reaction of the primary amino group of van with the phenyl ring of PDA (Fig. 2) [27]. SiO_2_-MNPs were similarly conjugated (Additional file 1: Fig. S1). TEM images showed that van-PDA-MNPs had a dark core covered with a transparent layer (Fig. 3a). The transparent layer was not observed in van-SiO_2_-MNPs (Additional file 1: Fig. S2a). The dark core and transparent layer indicate the presence of iron oxide and PDA. The diameter of the dark core and the thickness of the PDA layer were 97.7 ± 6.8 nm and 11.9 ± 1 nm (n = 5).

XPS analyses showed that van-PDA-MNPs were coated with PDA and grafted with van (Fig. 3b–d). MNPs were composed of Fe, O, and C (Fig. 3b)

. The presence of PDA on the MNPs was confirmed by the three peaks of O, N, and C, which are the elements of PDA (Fig. 3c). In van-PDA-MNPs, the characteristic peak of Cl2p for van was observed (Fig. 3d), indicating the grafting of the amine group of van onto the carbon ring of PDA [27]. However, the Fe peak disappeared in PDA-MNPs because PDA coating thicker than 10 nm blocked X-ray penetration into the MNPs [28].

The alteration in the surface charge of MNPs by PDA coating and van conjugation was analysed by measuring the zeta potential of each type of MNP. The surface charges of MNPs, PDA-MNPs, and van-PDA-MNPs were − 22.5 ± 1.6, − 13. 1 ± 0.7 and − 7.9 ± 0.7 mV, respectively, indicating that the negative charge of MNPs was reduced by the PDA coating and van conjugation (Fig. 3e). Coating of MNPs with SiO_2_ and conjugation of SiO_2_ with van also caused a reduction in the negative charge of MNPs (Additional file 1: Fig. S3). The results suggest that the relatively neutrally charged molecules such as PDA, SiO_2,_ and van compared to iron oxide nanoparticles can reduce the negative charge on the surface of MNPs when they are present on the surface.

The EDS mapping results showed a map of the elements (Fe, O, C and N) in each type of MNPs (Fig. 3f, Additional file 1: Fig. S2b), confirming the results obtained by the TEM images and XPS analyses. EDS mapping could not accurately reflect the presence of chlorine due to its low amount (about 1.5%) in van compared to the other elements (Table 1). Taken together, it is suggested that the MNPs were successfully coated with PDA and conjugated with van.

**Table 1:** The elemental composition of dried MNPs and their relative atomic concentration were studied by exciting the MNPs to mono-energetic Al kα x-rays and measuring the energy of photoelectrons emitted by electron energy analyser (ESCALAB250 XPS analyser)

Sample	Relative atomic mass (%)				
	Fe	O	C	N	Cl
MNPs	24.8	48.4	26.8	–	–
PDA-MNPs	–	24.0	69.5	6.5	–
Van-PDA-MNPs	–	23.3	65	10.2	1.5

### Particle size distribution in PBS and blood

**Fig. 4:**
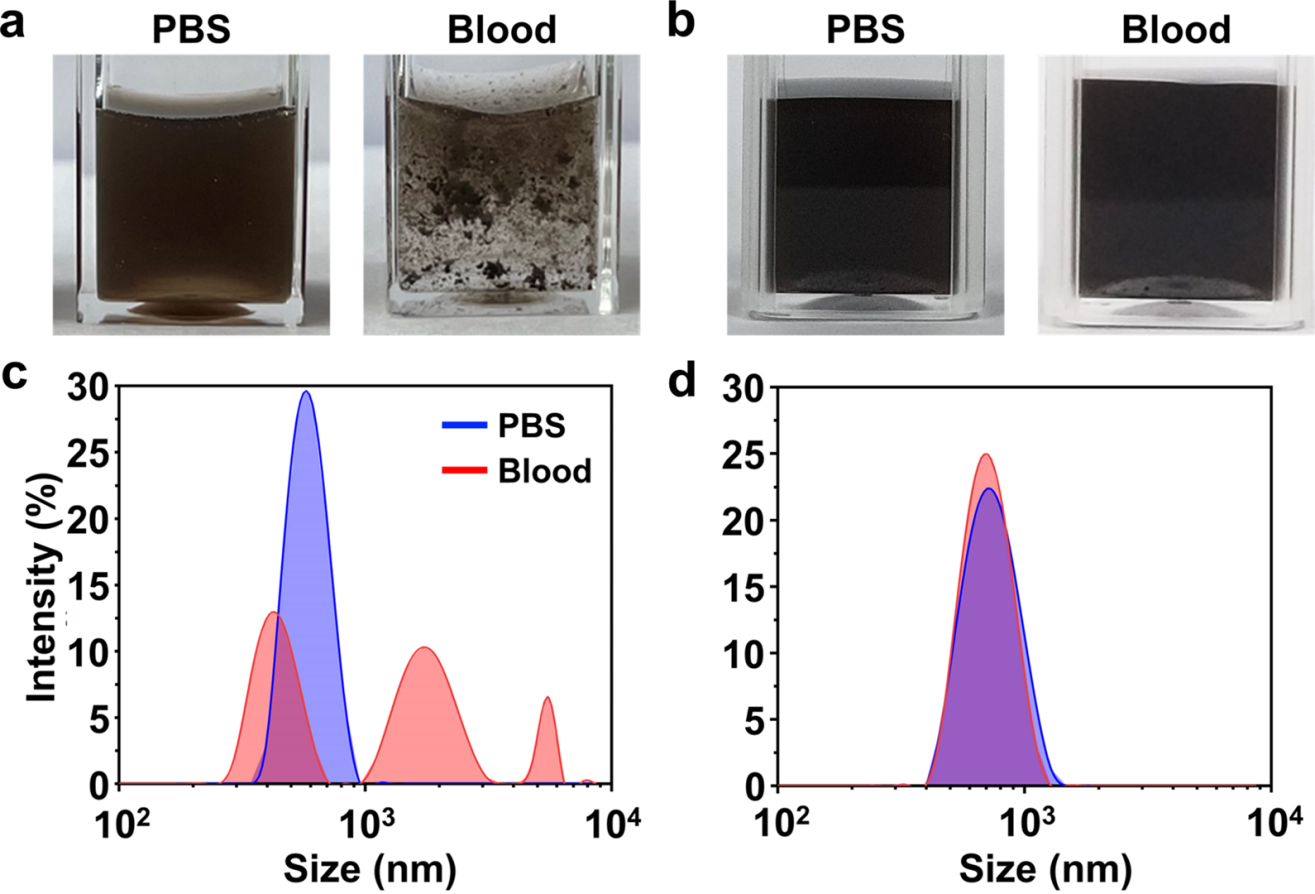
Dispersion of van-SiO_2_-MNPs and van-PDA-MNPs in PBS (**a**) and blood (**b**). The particle size distribution of the MNPs was analysed using Zetasizer. Two hundred microliters of each type of MNP were added to PBS or blood and incubated at 37 °C for 30 min. MNPs were then separated using a permanent magnet and washed with PBS, and the size distribution of van-SiO_2_-MNPs (**c**) and van-PDA-MNPs (**d**) was analysed

To test whether PDA coating can reduce the aggregation of MNPs in blood, the particle size of van-SiO_2_-MNPs and van-PDA-MNPs in PBS and blood was measured using Zetasizer Nano ZS (Malvern Instruments, Malvern, UK). Van-SiO_2_-MNPs did not aggregate in PBS but were severely aggregated in the blood (Fig. 4a, Additional file 1: Fig. S4). This is supported by the size distribution of van-SiO_2_-MNPs in PBS and blood, showing a single peak and three peaks, respectively (Fig. 4c). In contrast, van-PDA-MNPs did not aggregate in either PBS or blood (Fig. 4b), supported by the single peak in their size distribution (Fig. 4d). The silica oxide surface often generates a reactive oxygen species and interacts with biological membranes [14], promoting the non-specific adsorption of blood cells to the MNPs and the aggregation. In contrast, van-PDA-MNPs did not aggregate due to their strong hydrophilicity [16] that prevents blood cells and platelets from binding to the surface of PDA-MNPs. Our results suggest that the PDA coating could prevent the aggregation of MNPs in the blood.

### Preconcentration of different species of bacteria with van-PDA-MNPs

**Fig. 5:**
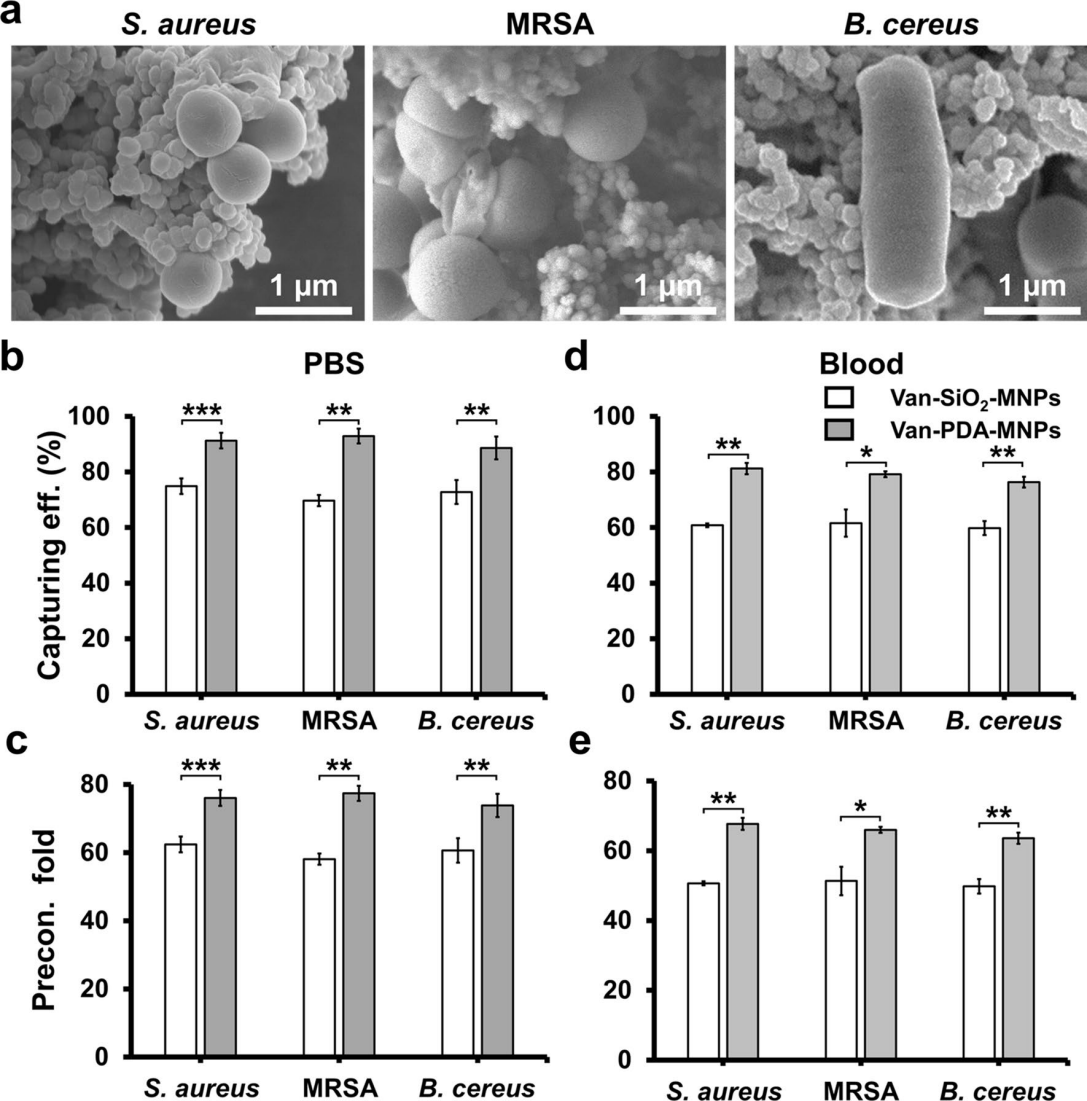
Binding of van-PDA-MNPs with bacteria. **a** SEM images of bacteria captured by van-PDA-MNPs. The capturing efficiency of van-PDA-MNPs and van-SiO_2_-MNPs for different species of bacteria in PBS (**b**) and blood (**c**) was analysed using the colony counting method. The capture efficiency was calculated based on the initial number of bacteria in the sample and the number of uncaptured bacteria cells in the eluent sample. Preconcentration fold in PBS (**d**) and blood (**e**). The preconcentration fold was estimated based on the capture efficiency and initial and final sample volume ratio. Student’s *t*-test, ***: *P* < 0.001. **: *P* < 0.01.*: *P* < 0.05. n = 3

Van-PDA-MNPs can bind to different strains (*S. aureus*, MRSA, and *Bacillus cereus*), which was verified by scanning electron microscopy (SEM) (Fig. 5a). It was previously reported that van nanoparticles could capture Gram-positive bacteria, including MRSA [24, 29, 30]. This is possible because van is a broad-spectrum antibiotic that can target the peptidoglycan layer of various species of Gram-positive bacteria [25, 26].

The capturing efficiencies of all the tested strains in PBS by van-SiO_2_-MNPs and van-PDA-MNPs were about 70 and 90% respectively. There is a significant difference (*P* < 0.001) in the capture efficiencies between the two types of MNPs (Fig. 5b). This leads to a difference in bacterial preconcentration capability between MNP types (Fig. 5c). The difference in the capturing efficiencies cannot be explained by the difference in cytotoxic effect between van-SiO_2_-MNPs and van-PDA-MNPs because van-SiO_2_-MNPs and van-PDA-MNPs did not negatively affect bacterial growth (Additional file 1: Fig. S5). XPS analysis showed that the atomic content of chlorine (Cl) derived from vancomycin hydrochloride on van-PDA-MNPs and van-SiO_2_-MNPs was 1.5% (Fig. 3d and Table 1) and 0.29% (Additional file 1: Fig. S6 and Table S1), respectively. This indicates that PDA coating increased the amount of van on the surface of PDA-MNPs. Zhao et al*.* [31] reported that PDA coating increased surface area and binding sites with amino and hydroxyl groups for receptors. Therefore, the PDA coating may increase the amount of van on the surface of PDA-MNPs. As a result, the bacteria capturing efficiency increased.

The capturing efficiency and preconcentration capability of van-PDA-MNPs in blood were also more significant than those of van-SiO_2_-MNPs (Fig. 5d, e). This might be due to the better compatibility of PDA coating with blood, reduced interaction and adhesion to blood cells and platelets [16]. As a result, it can easily capture the target bacteria without being blocked by blood cells. These results suggest that PDA coating eliminates the non-specific adsorption of blood cells onto MNPs and prevents the formation of aggregates.

### Improved molecular diagnostics for bacteria in the blood through preconcentration by van-PDA-MNPs

**Fig. 6:**
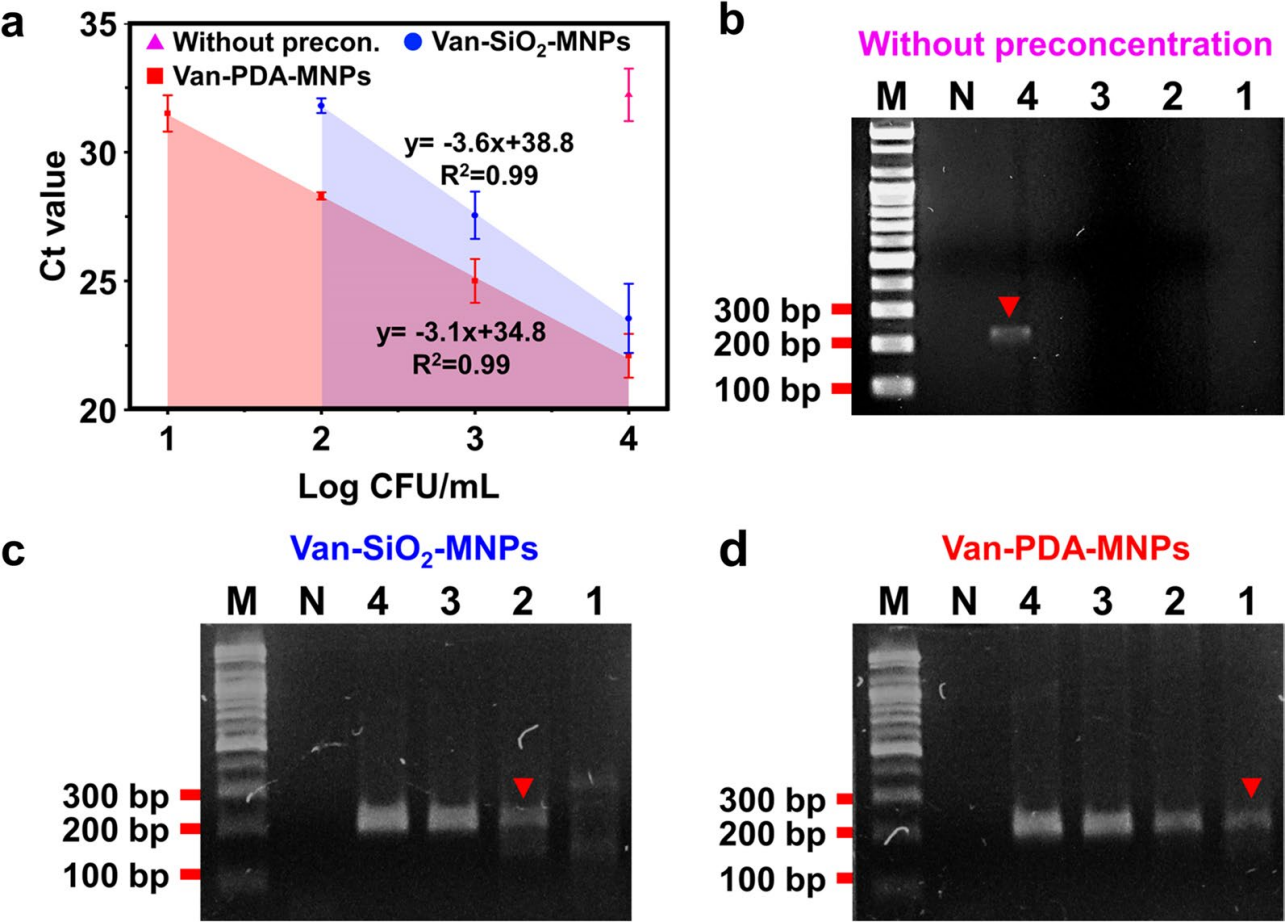
Improvement in the sensitivity of qPCR (**a**) and PCR with gel electrophoresis (**b**–**d**) to detect *S. aureus* in spiked blood samples through the preconcentration of the microorganism using van-PDA-MNPs. Ct value plotted against *S. aureus* at different concentrations (10^1^–10^4^ CFU/mL) in spiked blood samples with and without the preconcentration by van-SiO_2_-MNPs or van-PDA-MNPs. **b**–**d** Gel electrophoresis images of the amplified DNA (207 bp) of the target gene (*nuc*) by PCR with (**c**, **d**) and without (**b**) the preconcentration

qPCR was used to test whether preconcentration of bacteria by van-PDA-MNPs can enhance the sensitivity of molecular diagnostics for bacteria in the blood. DNA extracted from *S. aureus* in the blood by a genomic DNA extraction kit was used as a control without preconcentration by any MNP. Without the preconcentration, as low as 10^4^ CFU/mL was detectable (Fig. 6a). With either van-SiO_2_-MNPs or van-PDA-MNPs, as low as 10^2^ and 10 CFU/mL were detectable, respectively (Fig. 6a, Additional file 1: Fig. S7). A similar result was observed in PCR (Fig. 6b–d). The results showed that the bacterial concentration of van-SiO_2_-MNPs was not as good as that of van-PDA-MNPs for molecular diagnostics in the blood. This can be explained by Table 2, which shows that absorbance at 260/280 nm of purified DNA of bacteria preconcentrated by the former and latter beads was lower than and higher than 1.8, respectively. The absorbance ratio at 260/280 nm lower than 1.8 indicates protein contamination in the purified DNA. The contamination could occur due to the non-specific adsorption of blood cells to van-SiO_2_-MNPs, lowering extracted DNA concentration and PCR amplification. On the other hand, the absorbance ratio at 260/280 nm for DNA obtained after preconcentration with van-PDA-MNPs and DNA extraction shows high quality. Higher quality DNA was achieved as the PDA coating prevented the non-specific adsorption of blood cells to the MNPs. As a result, as low as 10 CFU/mL of *S. aureus* was detectable in the blood. In PBS, there was almost no difference in the sensitivity of qPCR for the pathogen between van-SiO_2_-MNPs and van-PDA-MNPs (Additional file 1: Fig. S8). The results suggest that the use of van-PDA-MNP eliminated the non-specific adsorption of blood cells and the formation of aggregates in the blood, which resulted in the successful removal of the PCR inhibitory compounds and improved LOD. As previously reported [23, 32], van and lectin conjugated with MNPs were used to magnetically separate bacteria from the blood. As the conventionally used MNPs interact with blood cells and form aggregates, they have included an extra step for treating whole blood to remove the blood cells through natural sedimentation [23] or centrifugation after they have lysed red blood cells [32]. Because of the ability of the PDA coating to prevent non-specific adsorption of blood cells and aggregation, van-PDA-MNPs can be used to capture and preconcentrate target bacteria from whole blood without the need for additional pretreatment steps.

**Table 2:** Yield and purity of genomic DNA extracted from *S. aureus* at various concentrations (10–10^4^ CFU/mL) in the blood through preconcentration with van-SiO_2_-MNPs and van-PDA-MNPs

CFU/mL	Van-SiO_2_-MNPs			Van-PDA-MNPs		
	Abs. (260/230)^1^	Abs. (260/280)^2^	DNA conc. (ng/µL)	Abs. (260/230)	Abs. (260/280)	DNA conc. (ng/µL)
10	1.9 ± 0.3	1.6 ± 0.2	17.1 ± 1.7	2.4 ± 0.1	1.9 ± 0.1	32.8 ± 0.6
10^2^	1.9 ± 0.1	1.5 ± 0.7	6.4 ± 0.7	2.2 ± 0	1.8 ± 0	37.4 ± 0.3
10^3^	1.8 ± 0	1.3 ± 0	10.9 ± 0.6	2.2 ± 0	1.6 ± 0.1	44.2 ± 1.2
10^4^	2.1 ± 0.3	1.7 ± 0.3	18.6 ± 0.3	2.2 ± 0.1	1.8 ± 0.1	64.4 ± 0.6

After preconcentration, bacterial DNA was extracted by using a kit (MagListo™ 5 M Genomic DNA extraction kit). DNA purity and concentration were determined by the ratio of absorbance (abs.) at 230, 260 and 280 nm by using a spectrophotometer (Nano-200)

^1^The absorbance ratio at 260 and 230 nm is used to assess the presence of contaminants such as phenol, that absorbs strongly at or near 230 nm

^2^The absorbance ratio at 260 and 280 nm is used to assess the presence of proteins and other contaminants that absorb strongly at or near 280 nm

### Multiplex preconcentration of *S. aureus*, MRSA and *B. cereus* in blood with van-PDA-MNPs for molecular diagnostics

**Fig. 7:**
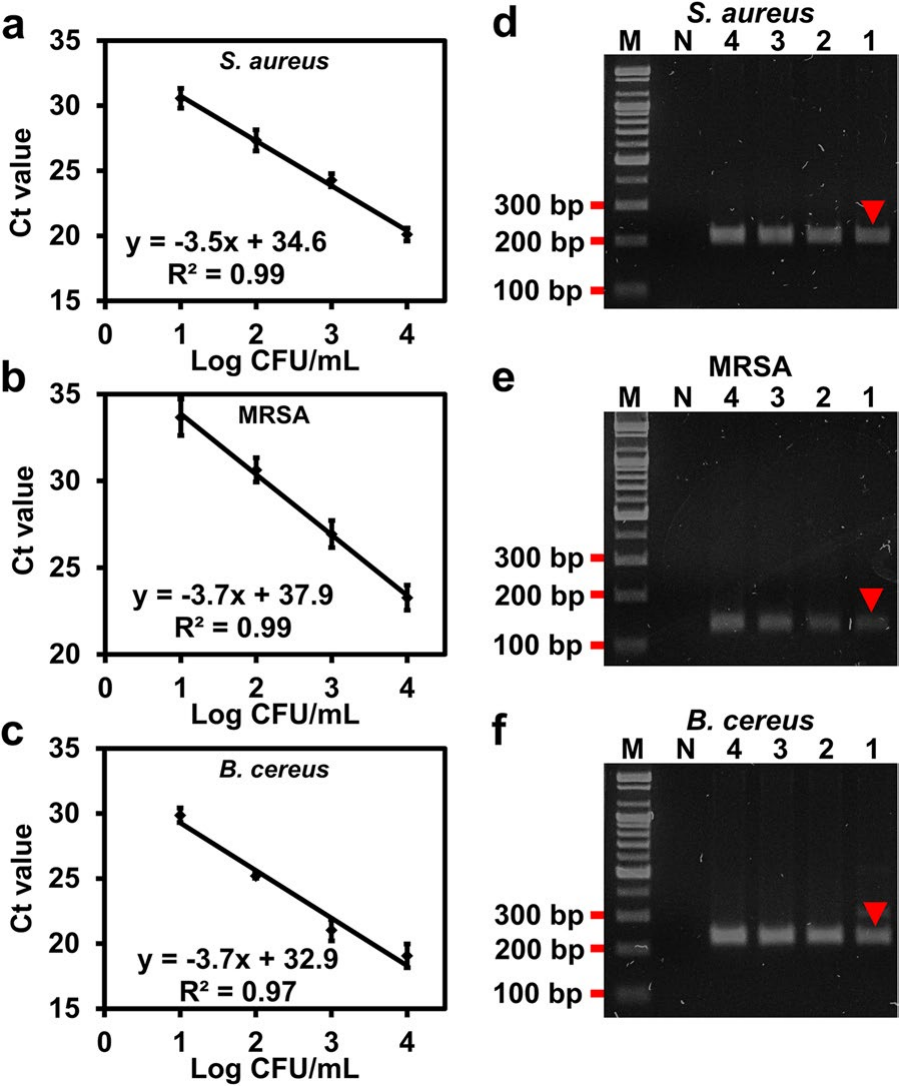
qPCR (**a**–**c**) and PCR with gel electrophoresis (**d**–**f**) for the detection of Gram-positive bacteria (*S. aureus* (a, d), MRSA (**b**, **e**), and *B. cereus* (**c**, **f**)) in the blood through the simultaneous preconcentration of the microorganisms using van-PDA-MNPs. The Materials and Method section contains details of each primer set and its target gene

qPCR and PCR were used to verify the performance of simultaneous preconcentration of several bacterial strains (*S. aureus*, MRSA, and *B. cereus*) in the blood with van-PDA-MNPs. All strains were detectable at as low as 10 CFU/mL by qPCR and PCR (Fig. 7, Additional file 1: Fig. S9). There was no significant difference in the preconcentration efficiencies for the three strains in PBS (Additional file 1: Fig. S10a) and blood (Additional file 1: Fig. S10b). This demonstrates that the use of van-PDA-MNPs prevented non-specific adsorption of blood cells and the formation of aggregates. Thus, van-PDA-MNPs can eliminate cumbersome pretreatment steps such as the separation of blood cells prior to the preconcentration of bacteria. As a result, the time required for preconcentration of pathogens can be reduced to 30 min.

## Conclusion

In this study, we synthesized MNPs coated with PDA and conjugated with van to separate multiple species of bacteria without aggregation of the MNPs. The LOD of molecular diagnostics for bacteria in blood samples through IMS with van-PDA-MNPs was 10 CFU/mL, which is close to culture-free detection. Since IMS with van-PDA-MNPs does not require prior sample pretreatment steps such as separating blood cells and dilution, bacterial preconcentration takes only 30 min. Van-PDA-MNPs can be used to preconcentrate only Gram-positive bacteria. However, their combined use of PDA-MNPs conjugated with polymyxin B, which targets Gram-negative bacteria, could enable simultaneous preconcentration of Gram-positive and Gram-negative bacteria in blood samples. On the other hand, antibodies conjugated to PDA-MNPs can be used to enrich and diagnose different viruses or isolate circulating tumour cells.

## Materials and methods

### Reagents

Ferric chloride hexahydrate, sodium acetate, ethylene glycol, 25% ammonia solution, 99% tetraethoxysilane (TEOS), (3-aminopropyl)triethoxysilane (APTES), 25% glutaraldehyde solution, sodium tetraborate, dopamine hydrochloride, vancomycin hydrochloride, oxacillin sodium salt and 99% osmium tetroxide (OsO_4_) were purchased from Sigma-Aldrich (St. Louis, MO, USA). Phosphate-buffered saline (PBS, pH 7.4) was purchased from Gibco (Grand Island, NY, USA).

### Bacterial culture

*S. aureus* (25923) and *B. cereus* (21722) were obtained from American Type Culture Collection (ATCC, Manassas, VA, USA). MRSA (3107) was obtained from Culture Collection of Antimicrobial Resistance Microbes (Seoul, Korea). A single colony of either *S. aureus* or *B. cereus* was transferred from the agar plate to 5 mL of Luria–Bertani (LB) broth (Becton, Dickinson and Company, Franklin Lakes, NJ, USA). LB broth supplemented with 6 µg/mL of oxacillin sodium salt was used to grow MRSA. Then, the culture was incubated overnight at 37 °C and 200 rpm. Fifty microliters of the overnight culture were transferred to a fresh 5 ml LB broth. Finally, it was incubated under the same conditions until the optical density (OD) measurement at 600 nm reached 1.

### Preparation of MNPs

Ferric chloride hexahydrate (0.54 g) and sodium acetate (0.5 g) were dissolved in 20 mL ethylene glycol with a continuous stirring [33]. The solution was then transferred to a 100-mL Teflon-lined hydrothermal autoclave and kept at 200 °C for 10 h. The autoclave was then kept at room temperature (RT) until it cooled down. The obtained MNPs were washed three times with 40 mL of deionized water and dried overnight. 100 mg of the MNPs were dispersed in 40 mL of HCL (1 M) and incubated at RT for 1 h under constant mixing. The MNPs were then collected using a permanent magnet and washed three times with PBS.

### Coating of MNPs with SiO_2_ and conjugation with van

0.5 mL of 25% ammonia solution and 0.2 mL of TEOS were added into 10 mL of deionized water containing 20 mg of MNPs in a sequential manner. The mixture was incubated for 24 h at RT [34]. MNPs were then washed three times with PBS (pH 7.4) and collected by using a permanent magnet. MNPs were then functionalized with an amine group by transferring 2 mg of SiO_2_-MNPs to a 1.5-mL tube containing 1 mL of 5% (3-aminopropyl) triethoxysilane (pH 4) and incubating at RT for 3 h. MNPs functionalized with the amine group were then mixed with 2.5% glutaraldehyde solution and incubated at RT for 30 min. The MNPs were then washed with 1 mL of 0.01 M borate buffer. Finally, 2 mg of van was added, and the mixture was incubated overnight. After washing three times with PBS, the MNPs were dispersed in 1 mL PBS.

### Coating of MNPs with PDA and conjugation with van

Fifty milligrams of MNPs were added to 25 mL of dopamine hydrochloride solution (2 mg/mL, pH 8.5) and incubated for 3 h under continuous stirring at RT [35]. PDA-MNPs were then separated by using a permanent magnet and washed three consecutive times with PBS. 25 mL of vancomycin hydrochloride solution (2 mg/mL, pH 7.4) was added to the PDA-MNPs and incubated at RT for 3 h (Fig. 2). Finally, MNPs were separated using a permanent magnet, washed three consecutive times with PBS and suspended in 25 mL PBS [34].

### Transmission electron microscopy (TEM) imaging

Two milligrams of the MNPs, PDA-MNPs and van-PDA-MNPs were washed three times with 1 mL DI water and suspended in 1 mL DI water. Ten microliters of the particles were then dropped onto a 300-mesh copper grid (CF-2/1-3CU-50) from Electron Microscopy Sciences (Hatfield, PA, USA) and dried at 70 °C for 2 h. Finally, morphology and elemental mapping of the particles were obtained at an accelerating voltage of 200kv by using JEM-2100F TEM (JEOL Ltd., Tokyo, Japan) and EDS attached to the TEM machine.

### XPS analysis

Dried MNPs were placed on a glass slide, and the XPS spectra of the samples were obtained by using ESCALAB250 XPS analyser (ThermoFisher Scientific, Waltham, MA, USA).

### Zeta potential measurement

Zeta potential measurements were performed by suspending 0.2 mg particles in 1 mL of DI water and analysing it with Zetasizer Nano ZS.

### Nanoparticle tracking and analysis

The concentration of particles was quantified by using NanoSight LM10 (Malvern Instruments, Malvern, UK).

### Particle size distribution in PBS and blood

Two hundred microliters of van-SiO_2_-MNPs or van-PDA-MNPs were added to 1 mL PBS or blood and incubated at 37 °C for 30 min. The MNPs were separated using a magnetic separation rack (MagListo™; Bioneer, Daejeon, Korea). MNPs were then washed three times with 1 mL PBS. Finally, they were suspended in 1 mL PBS and the size distribution of MNPs was analysed using Zetasizer.

### Scanning electron microscopy (SEM) imaging of bacteria captured by van-PDA-MNPs

Bacteria enriched with van-PDA-MNPs were washed twice with PBS and the MNPs were separated on a magnetic separation rack. Fixation of the bacteria-MNP complexes was done in 2% glutaraldehyde solution at RT for 1 h [36, 37]. Bacteria-MNP complexes were washed three times with 1 mL PBS and incubated in 1% osmium tetroxide for 1 h at 4 °C in the dark. Finally, after washing three times with PBS, gradual dehydration of the bacteria-MNP complexes was done in ethanol (30–100%) for 30 min each. Ten microliters of the bacteria-MNP complexes were dropped onto a 200-mesh copper grid (CF200-CU-50, Electron Microscopy Sciences) and dried at RT for 2 h. SEM observations were obtained by using JSM7500F SEM (JEOL Ltd.) with an accelerating voltage of 5 kV.

### Preconcentration in PBS and blood containing a single bacterial strain

1 mL of either PBS or blood containing 10^4^ of single bacterial strain (*S. aureus,* MRSA and *B. cereus)* CFU/mL was mixed with 200 µL of either van-PDA-MNPs or van-SiO_2_-MNPs and incubated at 37 °C for 30 min. Bacteria-MNP complexes were then separated using a magnetic separation rack, and the eluent was collected and inoculated on LB agar plates to perform standard colony counting. The eluent was grown in LB agar plates supplemented with 6 µg/mL oxacillin sodium salt to selectively grow MRSA. The colony numbers were used to calculate the number of uncaptured bacterial cells during the preconcentration. The following equation was used to calculate the capture efficiency of the MNPs for each species.

$$Capturing\,efficiency \left(\%\right)=\left(\frac{{N}_{t}-{N}_{u}}{{N}_{t}}\right)100\%$$

where *Nt* is the number of total bacteria cells in the sample and *Nu* is the number of uncaptured bacteria cells [38].

The preconcentration fold was calculated as follows [39].

$$Preconcentration\,fold=Capturing\, efficency*(\frac{Initial\,volume}{Preconcentrated\,volume})$$

### Preconcentration in blood containing multiple bacterial strains

First, 2.5 mL of blood containing three strains (*S. aureus*, MRSA and *B. cereus)* at different concentrations (10^1^–10^4^ CFU/mL) were mixed with 200 µL of either van-PDA-MNPs or van-SiO_2_-MNPs (10^11^ particles/mL, final concentration) [40]. Van-PDA-MNP at concentrations above 10^11^ particles/mL inhibited PCR (Additional file 1: Fig. S11). The mixture was incubated at 37 °C for 30 min. Bacteria-MNP complexes were separated using a magnetic rack and washed three times with 1 mL PBS.

### DNA extraction

Bacteria-particle complexes were then suspended in 200 µL PBS, and DNA extraction was performed using a commercialized DNA purification kit (MagListo™ 5 M Genomic DNA extraction kit, Bioneer, Korea). The purity and yield of the extracted DNA were determined based on the ratio of absorbance at wavelengths of 230, 260, and 280 nm using a spectrophotometer (Nano-200; AllSheng, Hangzhou City, China).

### PCR

Forward (5'-ACACCTGAAACAAAGCATCC-3') and reverse (5'-TAGCCAAGCCTTGACGAACT-3') primers were used to amplify 207 bp of *nuc* gene from purified *S. aureus* DNA [41]. Purified MRSA DNA was amplified by using the 135-bp *mecA* gene, forward (5'-AACCACCCAATTTGTCTGCC-3') and reverse (5'-TGATGGTATGCAACAAGTCGTAAA-3') primers [42]. Forward (5'-GCCCTGGTATGTATATTGGATCTAC-3') and reverse (5'-GGTCATAATAACTTCTACAGCAGGA-3') primers were used to amplify 220 bp of *gyrB* gene from purified *B. cereus* DNA [43]. MJ MINI thermocycler (Bio-RAD, Hercules, CA, USA) was used to perform PCR. PCR products were separated on a 2% TAE agarose gel at 100 V for 30 min.

### qPCR

The same primers were used for both PCR and qPCR. StepOne™ real-time PCR system (Applied Biosystems, Foster City, CA, USA) was used to perform qPCR.

### Statistical data analysis

The data shown are based on the mean ± standard deviation of three independently performed experiments. T-test was used to compare the data obtained under different conditions. Data with a p-value less than 0.05 were considered significant (*p < 0.05, **p < 0.01, ***p < 0.001).

## Supplementary Information

**Additional file 1. Table S1**. Elemental composition of dried van- SiO2-MNPs and their relative atomic concentration were studied by exciting the van- SiO2-MNPs to mono-energetic Al kα x-rays and measuring the energy of photoelectrons emitted by electron energy analyser (ESCALAB250 XPS analyser). **Fig. S1**. Schematic representing the synthesis of vancomycin-conjugated silica oxide-coated magnetic nanoparticles (van-SiO2-MNPs). Freshly prepared MNPs form larger MNPs through SiO2 coating. SiO2-MNPs are conjugated with van after amino group functionalization [1]. Tetraethyl orthosilicate (TEOS; Sigma-Aldrich). 3-Aminopropyltriethoxysilane (APTES; Sigma-Aldrich). www.biorender.com was used to make the schematics. **Fig. S2**. (a) Transmission electron microscopy (TEM) images and (b) energy dispersive spectroscopy (EDS) mapping of van-SiO2-MNPs. Morphology and elemental mapping of the particles were obtained at an accelerating voltage of 200kv by using JEM-2100F TEM (JEOL Ltd., Tokyo, Japan) and EDS attached to the TEM machine. **Fig. S3**. Zeta potential of MNPs, SiO2-MNPs and van-SiO2-MNPs. The potential was measured using Zetasizer Nano ZS (Malvern Instruments, Malvern, UK). Student’s t-test. **: P < 0.01. *: P < 0.05. n = 3. **Fig. S4**. Microscopic images of van- SiO2-MNPs and van-PDA-MNPs in blood. The images were taken using a DeltaVision microscope (GE Healthcare, Chicago, IL, USA). Before taking the images, blood was mixed with each type of MNPs at 1011 particles/mL (final conc.) and incubated in a rotary shaker for 30 min at RT. **Fig. S5**. Effect of van-PDA-MNPs and van- SiO2-MNPs on bacteria growth. Each strain was overnight cultured in LB broth with aeration (200 rpm) at 37 °C. Then, the culture was 100 times diluted with fresh LB broth containing either van-PDA-MNPs or van- SiO2-MNPs at 1011 particles/mL (final conc.). The diluted culture was incubated at the same growth condition and its optical density was measured at 600 nm. **Fig. S6**. Elemental composition of dried van- SiO2-MNPs and their relative atomic concentration were studied by exciting the van- SiO2-MNPs to mono-energetic Al kα x-rays and measuring the energy of photoelectrons emitted by electron energy analyser (ESCALAB250 XPS analyser). **Fig. S7**. (a) qPCR and (b) the standard curve (Ct value vs bacterial concentration) of S. aureus at different concentrations (101-104 CFU/mL) in PBS preconcentrated by van- SiO2-MNPs and van-PDA-MNPs. Forward (5'-ACACCTGAAACAAAGCATCC-3') and reverse (5'-TAGCCAAGCCTTGACGAACT-3') primers were used to amplify a 207-bp of nuc gene from S. aureus [2]. **Fig. S8**. Multiplex capturing efficiency of van-PDA-MNPs and van- SiO2-MNPs for different bacterial strains at 105 CFU/mL in PBS (a) and blood (b). The capture efficiency was calculated based on the initial number of bacteria in the sample and the number of uncaptured bacteria cells in the eluent sample. Bacterial numbers were counted using the standard colony counting method. Student’s t-test, *: P < 0.05. NS: P > 0.05. n = 3. **Fig. S9**. qPCR of different concentrations (101-104 CFU/mL) of S. aureus preconcentrated using van- SiO2-MNPs (a) and van-PDA-MNPs (b) in blood. **Fig. S10**. qPCR of different concentrations (101-104 CFU/mL) of three bacterial strains (S. aureus (a), MRSA (b) and B. cereus (c)) preconcentrated using van-PDA-MNPs in blood.

## Data Availability

Data sharing is not applicable to this article as no datasets were generated or analysed during the current study.

## Abbreviations

Ab: Antibody
Abs: Absorbance
APTES: (3-Aminopropyl)triethoxysilane
ATCC: American Type Culture Collection
*B. cereus*: *Bacillus cereus*
CCARM: Culture collection of antimicrobial-resistant microbes
CFU: Colony forming unit
EDS: Energy disruptive spectroscopy
Fe_3_O_4_: Iron oxide
IMS: Immunomagnetic separation
LB: Luria–Bertani
LOD: Limit of detection
mL: Millilitre
MNPs: Magnetic nanoparticles
MRSA: Methicillin-resistant *Staphylococcus aureus*
OD: Optical density
OsO4: Osmium tetroxide
PBS: Phosphate-buffered saline
PCR: Polymerase chain reaction
PDA: Polydopamine
qPCR: Quantitative PCR
*S. aureus*: *Staphylococcus aureus*
SEM: Scanning electron microscopy
SiO_2_: Silicon dioxide
TEM: Transmission electron microscopy
TEOS: Tetraethoxysilane
Van: Vancomycin
XPS: X-ray photoelectron spectroscopy
