# Supplementary material for: Vancomycin-conjugated polydopamine-coated magnetic nanoparticles for molecular diagnostics of Gram-positive bacteria in whole blood
Source: J Nanobiotechnology. 2022 Sep 5;20:400. doi: 10.1186/s12951-022-01606-3 (PMC9446563; doi:10.1186/s12951-022-01606-3)
Supplement: Supplementary file 1 — Additional file 1. Table S1. Elemental composition of dried van- SiO2-MNPs and their relative atomic concentration were studied by exciting the van- SiO2-MNPs to mono-energetic Al kα x-rays and measuring the energy of photoelectrons emitted by electron energy analyser (ESCALAB250 XPS analyser). Fig. S1. Schematic representing the synthesis of vancomycin-conjugated silica oxide-coated magnetic nanoparticles (van-SiO2-MNPs). Freshly prepared MNPs form larger MNPs through SiO2 coating. SiO2-MNPs are conjugated with van after amino group functionalization [1]. Tetraethyl orthosilicate (TEOS; Sigma-Aldrich). 3-Aminopropyltriethoxysilane (APTES; Sigma-Aldrich). www.biorender.com was used to make the schematics. Fig. S2. (a) Transmission electron microscopy (TEM) images and (b) energy dispersive spectroscopy (EDS) mapping of van-SiO2-MNPs. Morphology and elemental mapping of the particles were obtained at an accelerating voltage of 200kv by using JEM-2100F TEM (JEOL Ltd., Tokyo, Japan) and EDS attached to the TEM machine. Fig. S3. Zeta potential of MNPs, SiO2-MNPs and van-SiO2-MNPs. The potential was measured using Zetasizer Nano ZS (Malvern Instruments, Malvern, UK). Student’s t-test. **: P < 0.01. *: P < 0.05. n = 3. Fig. S4. Microscopic images of van- SiO2-MNPs and van-PDA-MNPs in blood. The images were taken using a DeltaVision microscope (GE Healthcare, Chicago, IL, USA). Before taking the images, blood was mixed with each type of MNPs at 1011 particles/mL (final conc.) and incubated in a rotary shaker for 30 min at RT. Fig. S5. Effect of van-PDA-MNPs and van- SiO2-MNPs on bacteria growth. Each strain was overnight cultured in LB broth with aeration (200 rpm) at 37 °C. Then, the culture was 100 times diluted with fresh LB broth containing either van-PDA-MNPs or van- SiO2-MNPs at 1011 particles/mL (final conc.). The diluted culture was incubated at the same growth condition and its optical density was measured at 600 nm. Fig. S6. Elemental composition of dried van- SiO [file 12951_2022_1606_MOESM1_ESM.docx]

**Additional file 1**

**Vancomycin-conjugated polydopamine-coated magnetic nanoparticles for molecular diagnostics of Gram-positive bacteria in whole blood**

Abdurhaman Teyib Abafogi^1+^, Tepeng Wu^2+^, Daekyu Lee^1^, Jinyeop Lee^1,2^, Gyoujin Cho^3^, Luke P. Lee^3,4,5^, Sungsu Park^1,3*^

^1^School of Mechanical Engineering, Sungkyunkwan University (SKKU), Suwon 16419, Korea

^2^KingoBio Inc., 31 Digital-ro 30-gil, Guro-gu, Seoul 08390, Korea

^3^Department of Biophysics, Institute of Quantum Biophysics, Sungkyunkwan University, Suwon, Korea

^4^Harvard Medical School, Department of Medicine, Brigham Women’s Hospital, Boston, MA, USA

^5^Department of Bioengineering, Department of Electrical Engineering and Computer Science, University of California at Berkeley, Berkeley, CA, USA

^+^Each author contributed equally to this work

^*^**Corresponding authors:** S. Park, School of Mechanical Engineering, Sungkyunkwan University (SKKU), Seobu-ro 2066, Jangan-gu, Suwon 16419, Korea. Tel: +82-31-290-7431. E-mail: nanopark@skku.edu

**Table S1.** Elemental composition of dried van- SiO_2_-MNPs and their relative atomic concentration were studied by exciting the van-SiO_2_-MNPs to mono-energetic Al kα x-rays and measuring the energy of photoelectrons emitted by electron energy analyser (ESCALAB250 XPS analyser).

| Name | Relative atomic mass (%) |
| --- | --- |
| O | 67.68 |
| N | 2.53 |
| Si | 16.65 |
| Cl | 0.29 |
| Fe | 12.85 |


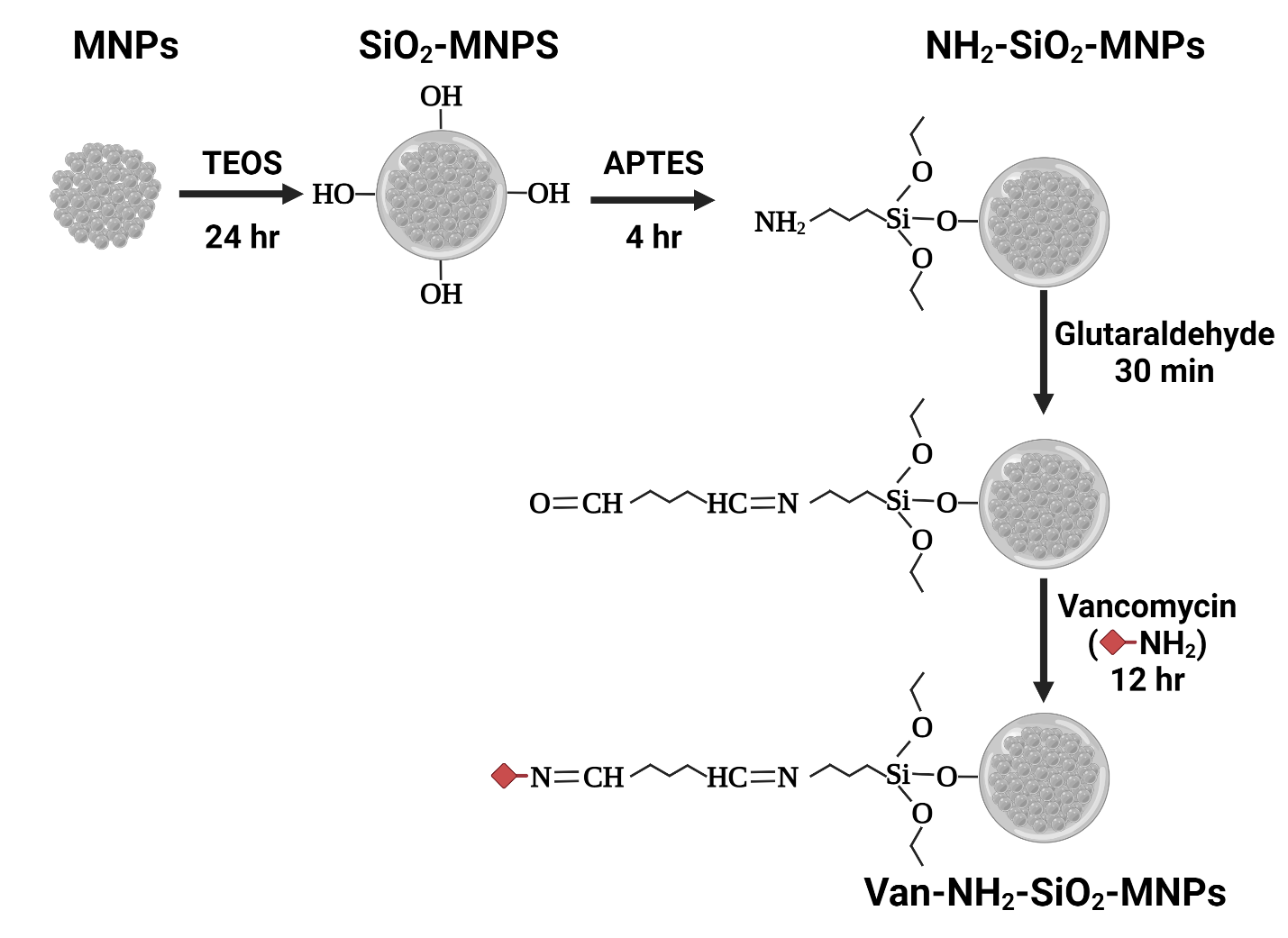


**Fig. S1.** Schematic representing the synthesis of vancomycin-conjugated silica oxide-coated magnetic nanoparticles (van-SiO_2_-MNPs). Freshly prepared MNPs form larger MNPs through SiO_2_ coating. SiO_2_-MNPs are conjugated with van after amino group functionalization [1]. Tetraethyl orthosilicate (TEOS; Sigma-Aldrich). 3-Aminopropyltriethoxysilane (APTES; Sigma-Aldrich). www.biorender.com was used to make the schematics.


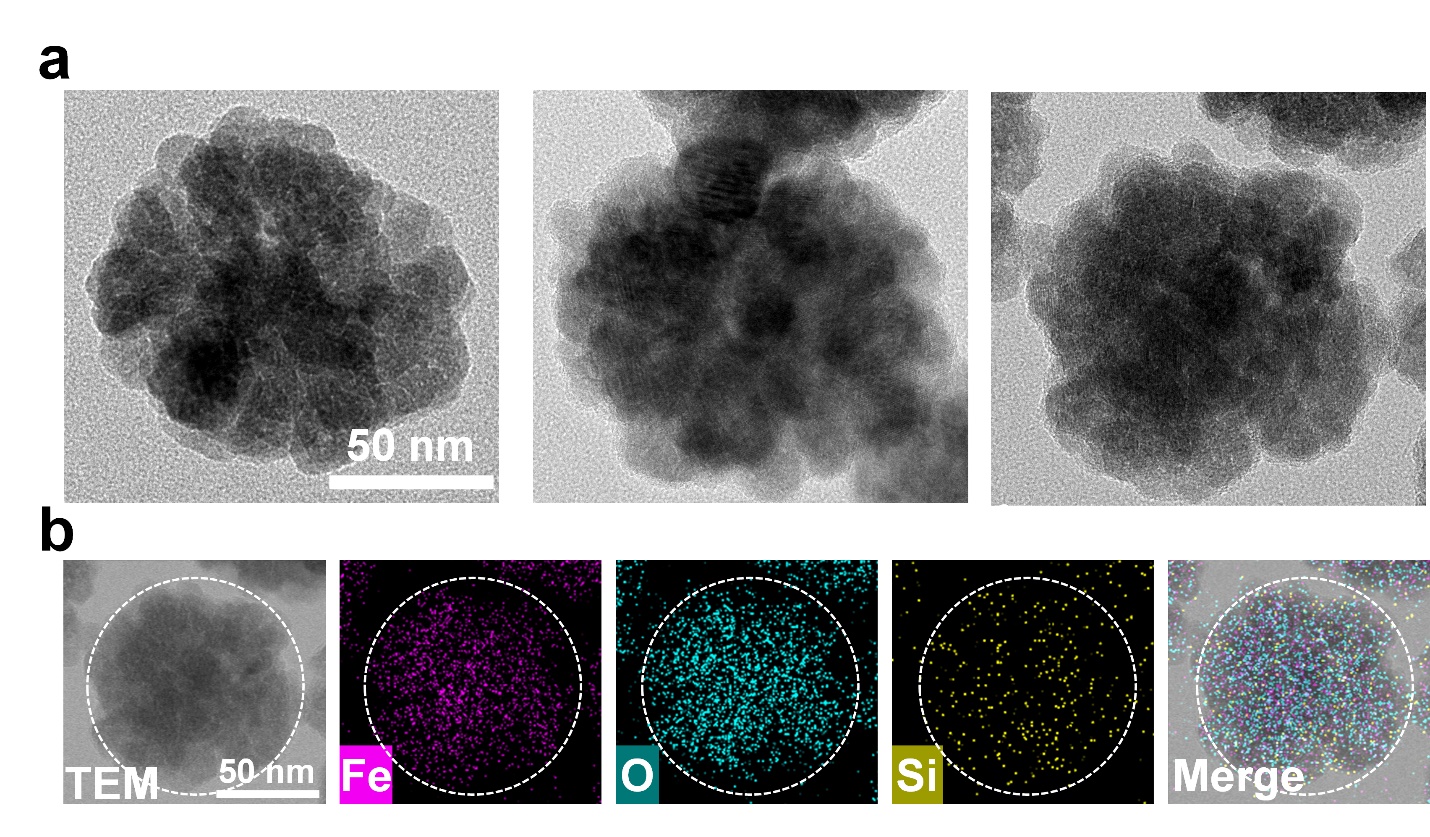
**Fig. S2.** **(a)** Transmission electron microscopy (TEM) images and **(b)** [energy dispersive spectroscopy (EDS) mapping of van-SiO](https://www.thermofisher.com/kr/ko/home/materials-science/eds-technology.html)_[2](https://www.thermofisher.com/kr/ko/home/materials-science/eds-technology.html)_[-MNPs. Morphology and elemental mapping of the particles were obtained at an accelerating voltage of 200kv by using JEM-2100F TEM (JEOL Ltd., Tokyo, Japan) and EDS attached to the TEM machine.](https://www.thermofisher.com/kr/ko/home/materials-science/eds-technology.html)


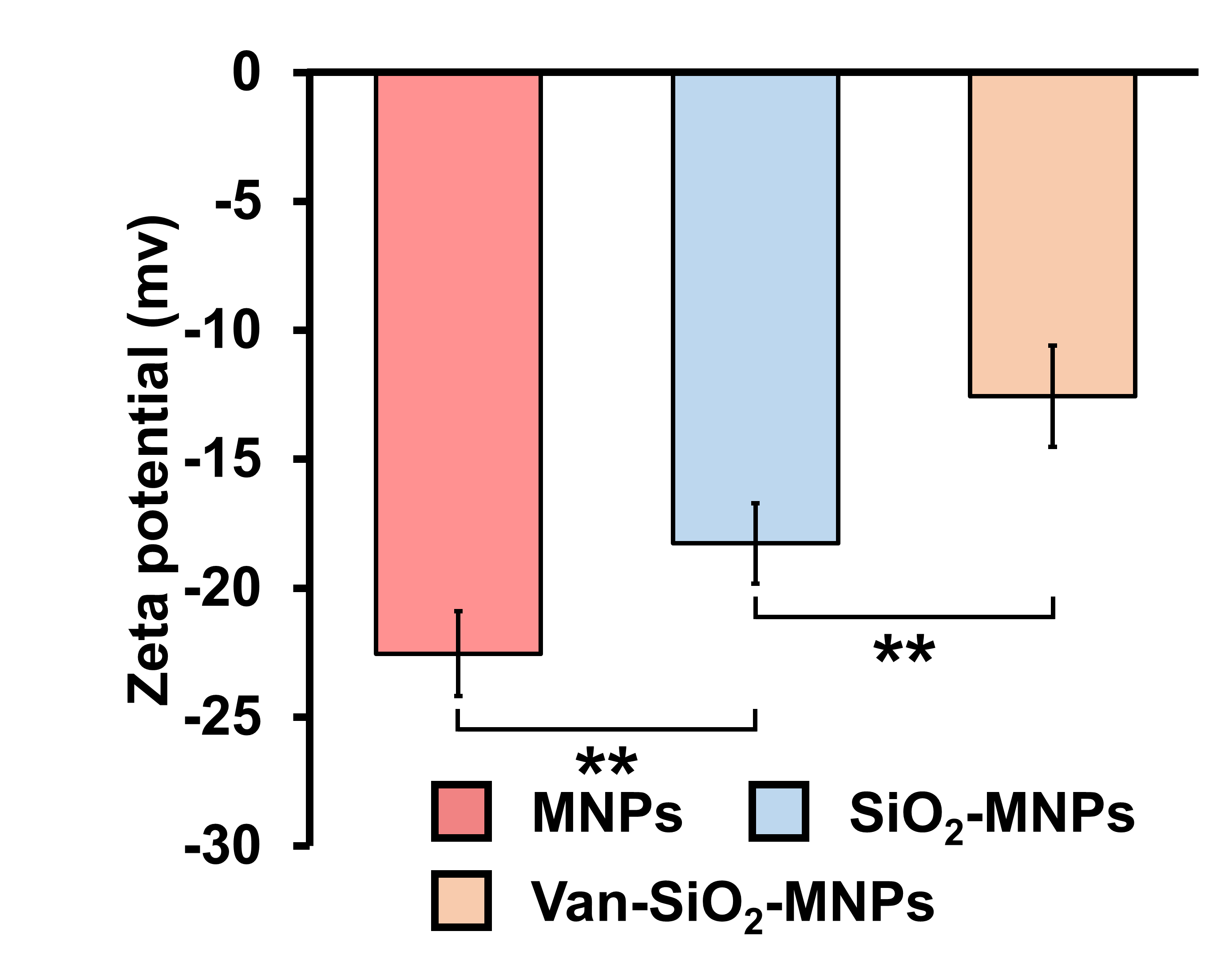


**Fig. S3.** Zeta potential of MNPs, SiO_2_-MNPs and van-SiO_2_-MNPs. The potential was measured using Zetasizer Nano ZS (Malvern Instruments, Malvern, UK). Student’s *t*-test. **: *P* < 0.01. *: *P* < 0.05. n = 3.


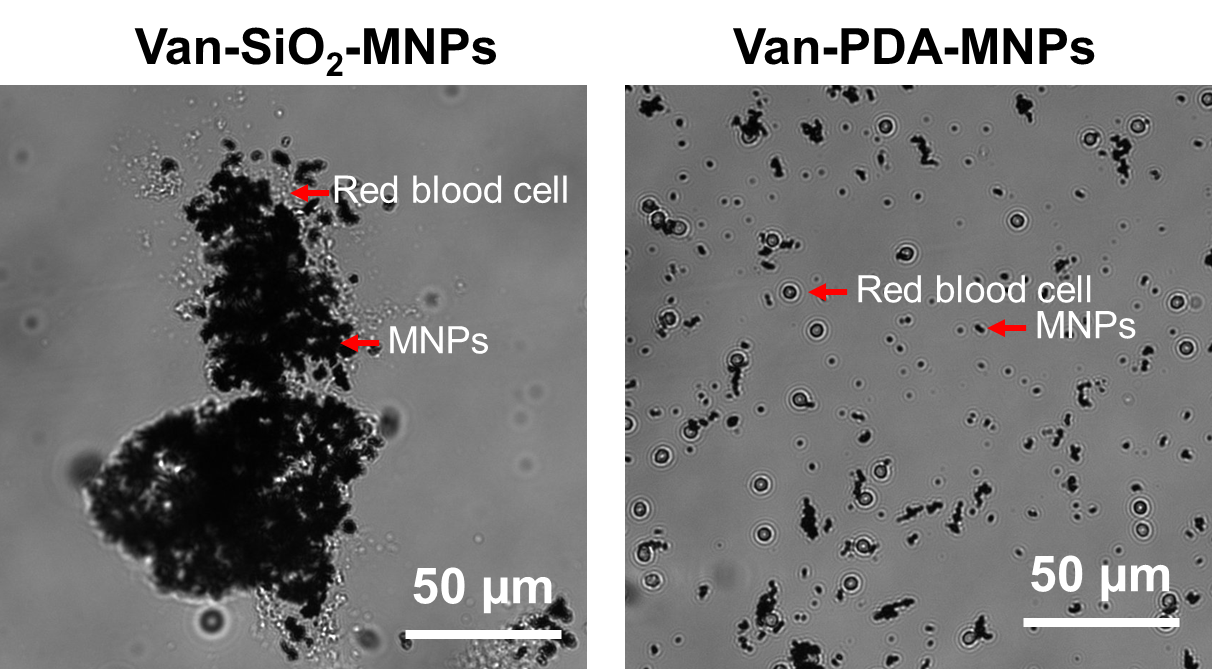


**Fig. S4.** Microscopic images of van- SiO_2_-MNPs and van-PDA-MNPs in blood. The images were taken using a DeltaVision microscope (GE Healthcare, Chicago, IL, USA). Before taking the images, blood was mixed with each type of MNPs at 10^11^ particles/mL (final conc.) and incubated in a rotary shaker for 30 min at RT.

**
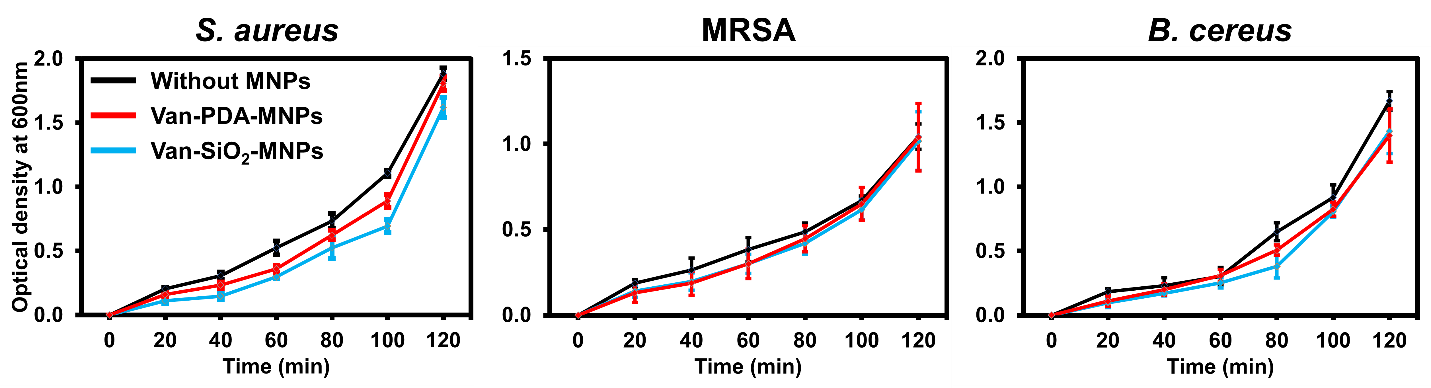
**

**Fig. S5.** Effect of van-PDA-MNPs and van- SiO_2_-MNPs on bacteria growth. Each strain was overnight cultured in LB broth with aeration (200 rpm) at 37 °C. Then, the culture was 100 times diluted with fresh LB broth containing either van-PDA-MNPs or van- SiO_2_-MNPs at 10^11^ particles/mL (final conc.). The diluted culture was incubated at the same growth condition and its optical density was measured at 600 nm.


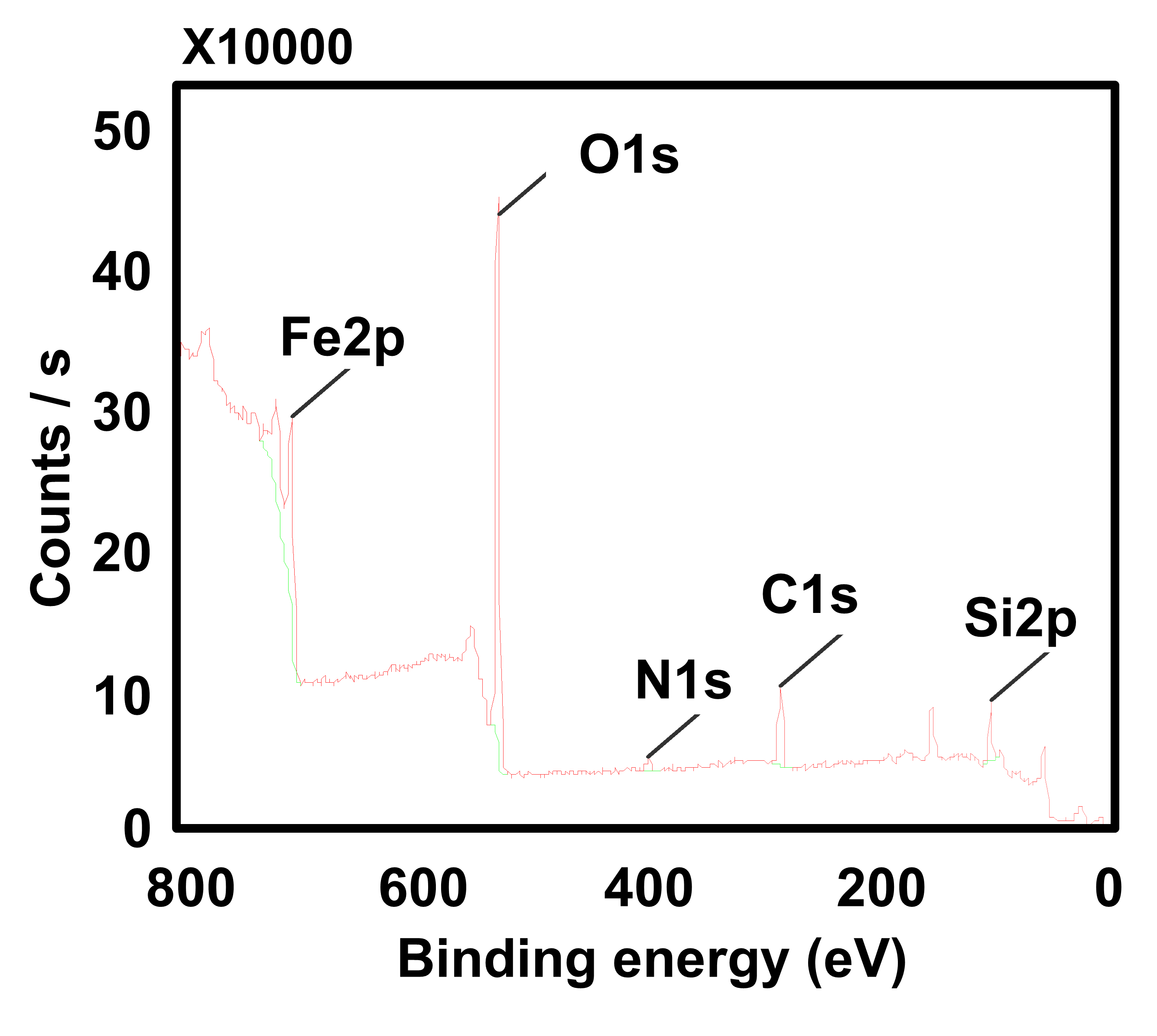


**Fig. S6.** Elemental composition of dried van- SiO_2_-MNPs and their relative atomic concentration were studied by exciting the van- SiO_2_-MNPs to mono-energetic Al kα x-rays and measuring the energy of photoelectrons emitted by electron energy analyser (ESCALAB250 XPS analyser)


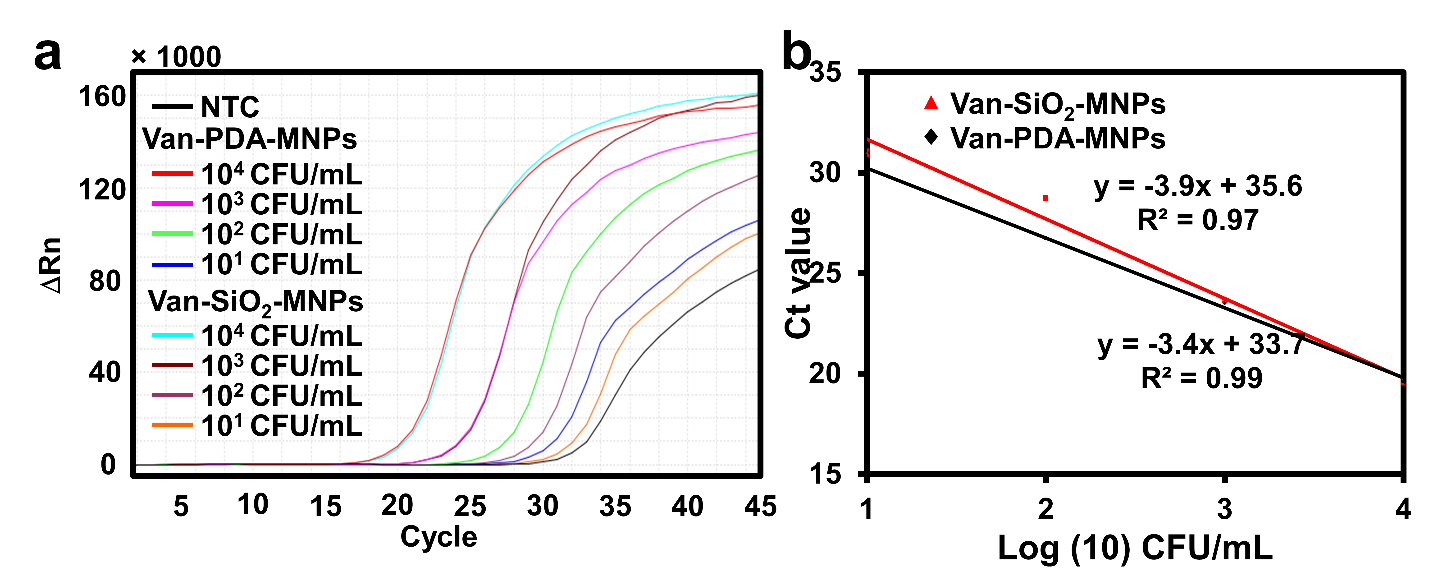


**Fig. S7. (a)** qPCR and **(b)** the standard curve (Ct value vs bacterial concentration) of *S. aureus* at different concentrations (10^1^-10^4^ CFU/mL) in PBS preconcentrated by van- SiO_2_-MNPs and van-PDA-MNPs. Forward (5'-ACACCTGAAACAAAGCATCC-3') and reverse (5'-TAGCCAAGCCTTGACGAACT-3') primers were used to amplify a 207-bp of *nuc* gene from *S. aureus* [2].


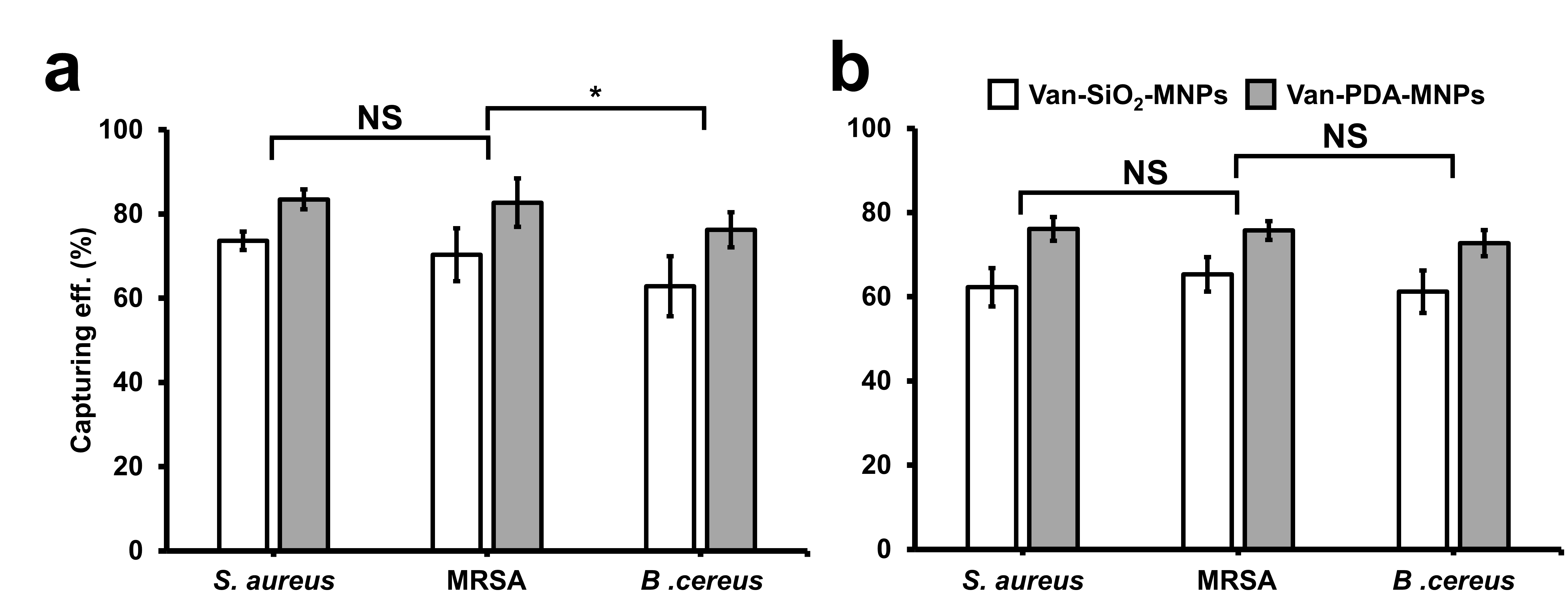


**Fig. S8.** Multiplex capturing efficiency of van-PDA-MNPs and van- SiO_2_-MNPs for different bacterial strains at 10^5^ CFU/mL in PBS **(a)** and blood **(b)**. The capture efficiency was calculated based on the initial number of bacteria in the sample and the number of uncaptured bacteria cells in the eluent sample. Bacterial numbers were counted using the standard colony counting method. Student’s *t*-test, *: *P* < 0.05. NS: *P* > 0.05. n = 3.


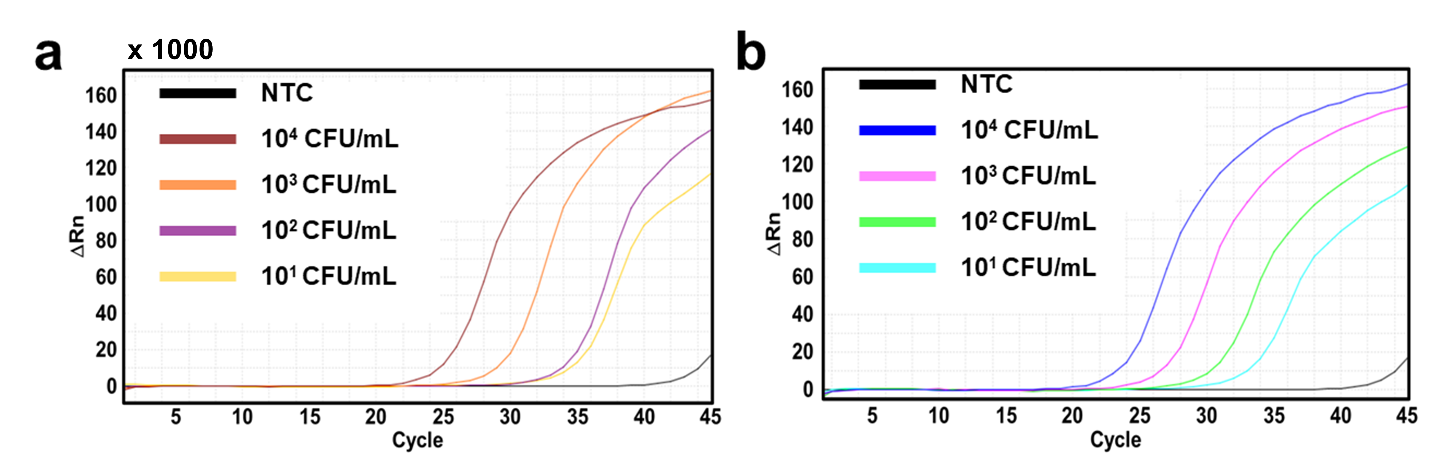


**Fig. S9.** qPCR of different concentrations (10^1^-10^4^ CFU/mL) of *S. aureus* preconcentrated using van- SiO_2_-MNPs (a) and van-PDA-MNPs (b) in blood.


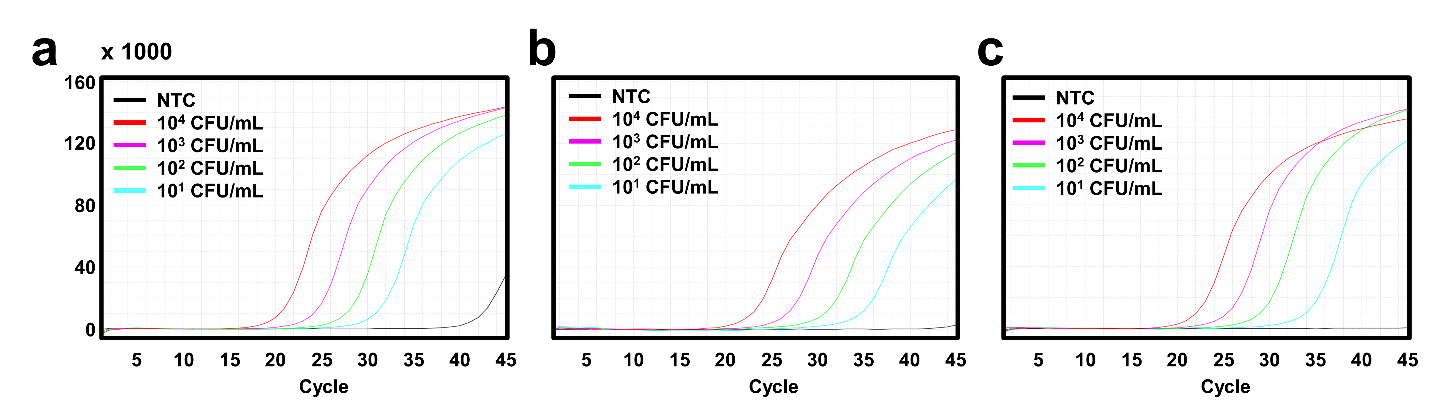


**Fig. S10.** qPCR of different concentrations (10^1^-10^4^ CFU/mL) of three bacterial strains (*S.* *aureus* (a), MRSA (b) and *B. cereus* (c)) preconcentrated using van-PDA-MNPs in blood.


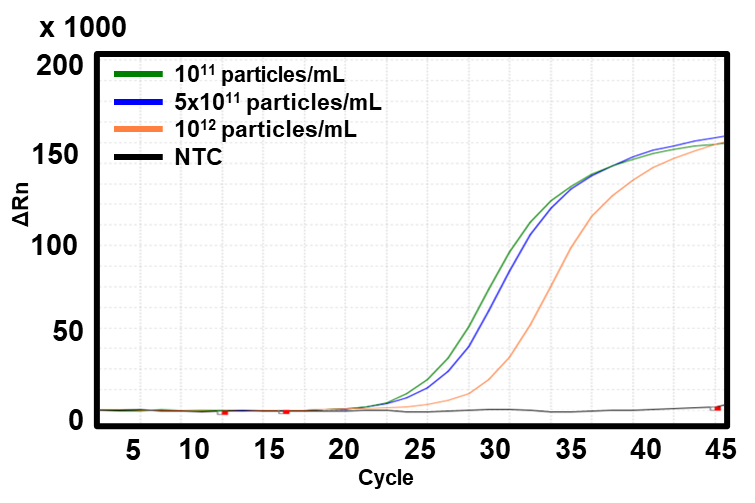


**Fig. S11.** qPCR result for *S. aureus* preconcentrated with various concentrations (10^11^-10^12^ particles/mL) of van-PDA-MNPs in blood.

**References**

1. P.Y. Furlan, A.Y. Furlan, K. Kisslinger, M.E. Melcer, D.W. Shinn, J.B. Warren. Water as the solvent in the stober process for forming ultrafine silica shells on magnetite nanoparticles. ACS 492 Sustain. Chem. Eng. 2019; 7: 15578-15584

2. L.E. O'Donnell, K. Smith, C. Williams, C.J. Nile, D.F. Lappin, D. Bradshaw, M. Lambert, D.P. Robertson, J. Bagg, V. Hannah. Dentures are a reservoir for respiratory pathogens. J. Prosthodont. 2016; 25: 99-104.
